# Identification of hub genes based on integrated analysis of single-cell and microarray transcriptome in patients with pulmonary arterial hypertension

**DOI:** 10.1186/s12864-023-09892-3

**Published:** 2023-12-18

**Authors:** Yuhan Qin, Gaoliang Yan, Yong Qiao, Dong Wang, Chengchun Tang

**Affiliations:** Department of Cardiology, Zhongda Hospital, School of Medicine, Southeast University, Nanjing, Jiangsu 210009 People’s Republic of China

**Keywords:** Hub gene, WDR43, GNL42, Pulmonary arterial hypertension, Single-cell sequencing, GEO

## Abstract

**Background:**

Pulmonary arterial hypertension (PAH) is a devastating chronic cardiopulmonary disease without an effective therapeutic approach. The underlying molecular mechanism of PAH remains largely unexplored at single-cell resolution.

**Methods:**

Single-cell RNA sequencing (scRNA-seq) data from the Gene Expression Omnibus (GEO) database (GSE210248) was included and analyzed comprehensively. Additionally, microarray transcriptome data including 15 lung tissue from PAH patients and 11 normal samples (GSE113439) was also obtained. Seurat R package was applied to process scRNA-seq data. Uniform manifold approximation and projection (UMAP) was utilized for dimensionality reduction and cluster identification, and the SingleR package was performed for cell annotation. FindAllMarkers analysis and ClusterProfiler package were applied to identify differentially expressed genes (DEGs) for each cluster in GSE210248 and GSE113439, respectively. Gene Ontology (GO) and Kyoto Encyclopedia of Genes and Genome (KEGG) were used for functional enrichment analysis of DEGs. Microenvironment Cell Populations counter (MCP counter) was applied to evaluate the immune cell infiltration. STRING was used to construct a protein-protein interaction (PPI) network of DEGs, followed by hub genes selection through Cytoscape software and Veen Diagram.

**Results:**

Nineteen thousand five hundred seventy-six cells from 3 donors and 21,896 cells from 3 PAH patients remained for subsequent analysis after filtration. A total of 42 cell clusters were identified through UMAP and annotated by the SingleR package. 10 cell clusters with the top 10 cell amounts were selected for consequent analysis. Compared with the control group, the proportion of adipocytes and fibroblasts was significantly reduced, while CD8+ T cells and macrophages were notably increased in the PAH group. MCP counter revealed decreased distribution of CD8+ T cells, cytotoxic lymphocytes, and NK cells, as well as increased infiltration of monocytic lineage in PAH lung samples. Among 997 DEGs in GSE113439, module 1 with 68 critical genes was screened out through the MCODE plug-in in Cytoscape software. The top 20 DEGs in each cluster of GSE210248 were filtered out by the Cytohubba plug-in using the MCC method. Eventually, WDR43 and GNL2 were found significantly increased in PAH and identified as the hub genes after overlapping these DEGs from GSE210248 and GSE113439.

**Conclusion:**

WDR43 and GNL2 might provide novel insight into revealing the new molecular mechanisms and potential therapeutic targets for PAH.

## Introduction

Pulmonary artery hypertension (PAH) is a chronic severe progressive cardiopulmonary disease characterized by pulmonary arterial pressure elevation and right ventricular hypertrophy [1]. The prevalence of PAH is 10.6 per million adults in America nowadays [2]. Despite the benefits of treatments targeting nitric oxide, prostacyclin, and endothelin pathways to delayPAH progression and improve survival, only lung transplantation is considered a curative approach [3]. PAH remains an incurable chronic disease with a poor prognosis [4]. Vasoconstriction, obstructive pulmonary vasculopathy characterized by hyperproliferation and anti-apoptosis phenotype of PASMCs, excessive fibrosis, inflammation, thrombosis, and altered mitochondrial metabolic all participated in the mechanisms implicated in PAH [5]. However, there remains largely unexplored on the pathogenesis of PAH. Therefore, systematic analysis of the function of different cell types in the pulmonary tissue of PAH patients might help deepen understanding of the pathological mechanism of PAH.

Microarray transcriptome has been increasingly and widely used to examine gene expression in PAH [6, 7]. However, data of microarray transcriptome represents the average gene expression amounts of various cells at the whole level of tissue [8]. Lung tissues contain various cell types, including smooth muscle cells, endothelial cells, fibroblasts, immune cells, inflammatory cells, etc. They play different roles throughout the development of PAH. Currently, a novel single-cell RNA sequencing (scRNA-seq) technology is emerging to investigate cell heterogeneity, characterize each cell subpopulation, and putative intracellular communication [9, 10]. This innovative technology has advanced our understanding of PAH at the cell subpopulation level. scRNA-seq has been carried out in lung samples of both PAH rodent models and PAH patients. Previous research reported NF-κB signaling activation in immune cells of monocrotaline and hypoxia-induced PH rat model [11]. Based on the scRNA-seq data of lung ECs from hypoxic pulmonary hypertension mice, Julie and his colleagues indicated CD74 was involved in the regulation of endothelial cell proliferation and barrier integrity [12]. However, scRNA-seq data on PAH is relatively small and still in its infancy currently.

In the present study, integrated bioinformatics analysis of scRNA-seq and microarray transcriptome data from the GEO dataset was analyzed to identify the hub genes in PAH. Differentially expressed genes (DEGs) from GSE210248 and GSE113439 were identified and common DEGs were selected. Protein-protein interaction network (PPI) network was constructed using the aforementioned DEGs, followed by hub gene selection through Cytoscape software. Finally, GNL2 and WDR43 were identified as hub genes, which might provide new insight into the pathogenesis of PAH and act as novel candidates and therapeutic targets for PAH.

## Materials and methods

### Data acquisition

Data were all processed and analyzed by R software (Version 4.3.0). Both scRNA-seq (GSE210248) and microarray transcriptome (GSE113439) data were obtained from the Gene Expression Omnibus (GEO, http://www.ncbi.nlm.nih.gov/geo/) database [13] and downloaded through the GEO query package (Version 2.68.0). GSE210248 and GSE113439 were selected in the current research because the samples in the two datasets were obtained from the lung/pulmonary arteries of participants with pulmonary hypertension, rather than the PAH rodent model. Additionally, GSE11339 has a relatively large The details of the two datasets enrolled in this study were listed in Table 1. The GSE210248 scRNA-seq data and GSE113439 array data were generated on GPL20301 Illumina Hiseq 4000 and GPL6244 Affymetrix Human Gene 1.0 ST Array platform, respectively. GSE210248 data included pulmonary arteries from 3 PAH patients and 3 healthy donor control. The dataset contains 19,576 cells from the control group and 22,704 cells from the PAH group. The data of GSE113439 included fresh frozen lung samples from the recipients’ organs of 15 PAH patients and 11 normal lung samples obtained from tissue flanking lung cancer resections.

**Table 1:** Overview of the enrolled datasets in the current study

Datasets	Type	Platform	Sample size (PAH/Control)	Cells (Control/HPH)
GSE210248	scRNA sequencing	GPL20301 Illumina Hiseq 4000	3/3	19,576/22704
GSE113439	microarray	GPL6244 Affymetrix Human Gene 1.0 ST Array	15/11	-

### Processing of scRNA-seq data

Seurat package (Version 4.3.0) was used for quality control. Cells with 200–2500 genes and < 5% mitochondrial genes were selected for consequent analysis. A total of the 19,576 cells in control group and 21,896 cells in the PAH group were screened out for analysis. Data of genes was further normalized using the “LogNormalize” method and further scaled. Then, the top 2000 highly variable genes (HVGs) were identified by the FindVariableFetures function with the “vst” method. Subsequently, principal component analysis (PCA) was applied to identify significant principal components (PCs), and the *p*-value was visualized using the JackStraw and ScoreJackStraw functions. Uniform manifold approximation and projection (UMAP) was utilized for dimensionality reduction with 20 PCs and cluster identification across these cells. “Harmony” R package was used for batch correction to avoid the batch effect of sample identity which might disrupt the downstream analysis [14]. SingleR package (Version 2.2.0) [15] was utilized for cell annotation according to the reference datasets HumanPrimaryCellAtlasData [16] and BlueprintEncodeData [17]. FindAllMarkers analysis with |log_2_ fold change (FC)|> 1 and adjusted *p* value < 0.05 were performed to screen out the differentially expressed genes (DEGs) for each cell cluster. scRNAtoolVis package (Version 0.0.5) was performed to display the top DEGs and visualized by jjvolcano.

### Processing of microarray transcriptome data

DEGs between the control and PAH groups with an adjusted *p* value < 0.05 were screened out using the limma package (Version 3.56.2) [18]. All DEGs were visualized using the volcano plot and the top 50 DEGs were visualized through the heatmap plot in the “ggplot2” package.

### Functional enrichment analysis

Gene Ontology (GO) [19] and Kyoto Encyclopedia of Genes and Genomes (KEGG) [20] analysis were carried out by the clusterprofiler package [21]. GO enrichment included 3 subontologies: biological process (BP), molecular function (MF), and cellular component (CC) [19]. *P* < 0.05 is considered statistically significant.

### Microenvironment Cell Populations counter (MCP counter)

The infiltration of microenvironment immune cells including B lineage, CD8 T cells, cytotoxic lymphocytes, endothelial cells, monocytic lineage, myeloid dendritic cells, neutrophils, NK cells, and T cells was quantified through the MCP counter (Version 1.2.0) based on scRNA-seq data [22].

### PPI network construction and identification of hub genes

DEGs in scRNA-seq and microarray transcriptome data were screened by FindAllMarkers analysis in the Seurat package and the limma package. Subsequently, protein–protein interaction (PPI) networks were constructed for the prediction of internal connection among the picked DEGs using the STRING database (Version 11.5, https://string-db.org/) with an interaction conference score set to 0.4 [23]. Then, hub genes were screened out and network visualization was performed using Cytoscape software (Version 3.10.0) [24]. The Molecular Complex Detection (MCODE) plug-in was used to build clustering function modules in the PPI network. Then, the CentiScaPe plug-in was used to calculate the degree, betweenness, and centroid value of each gene within the network. CytoHubba plug-in was used for ranking nodes in the target network using Maximal Clique Centrality (MCC) methods. The Venn diagram was produced by the jvenn website (https://jvenn.toulouse.inrae.fr/app/example.html) for gene overlapping and common gene selection.

## Results

### ScRNA profiling in PAH

**Fig. 1:**
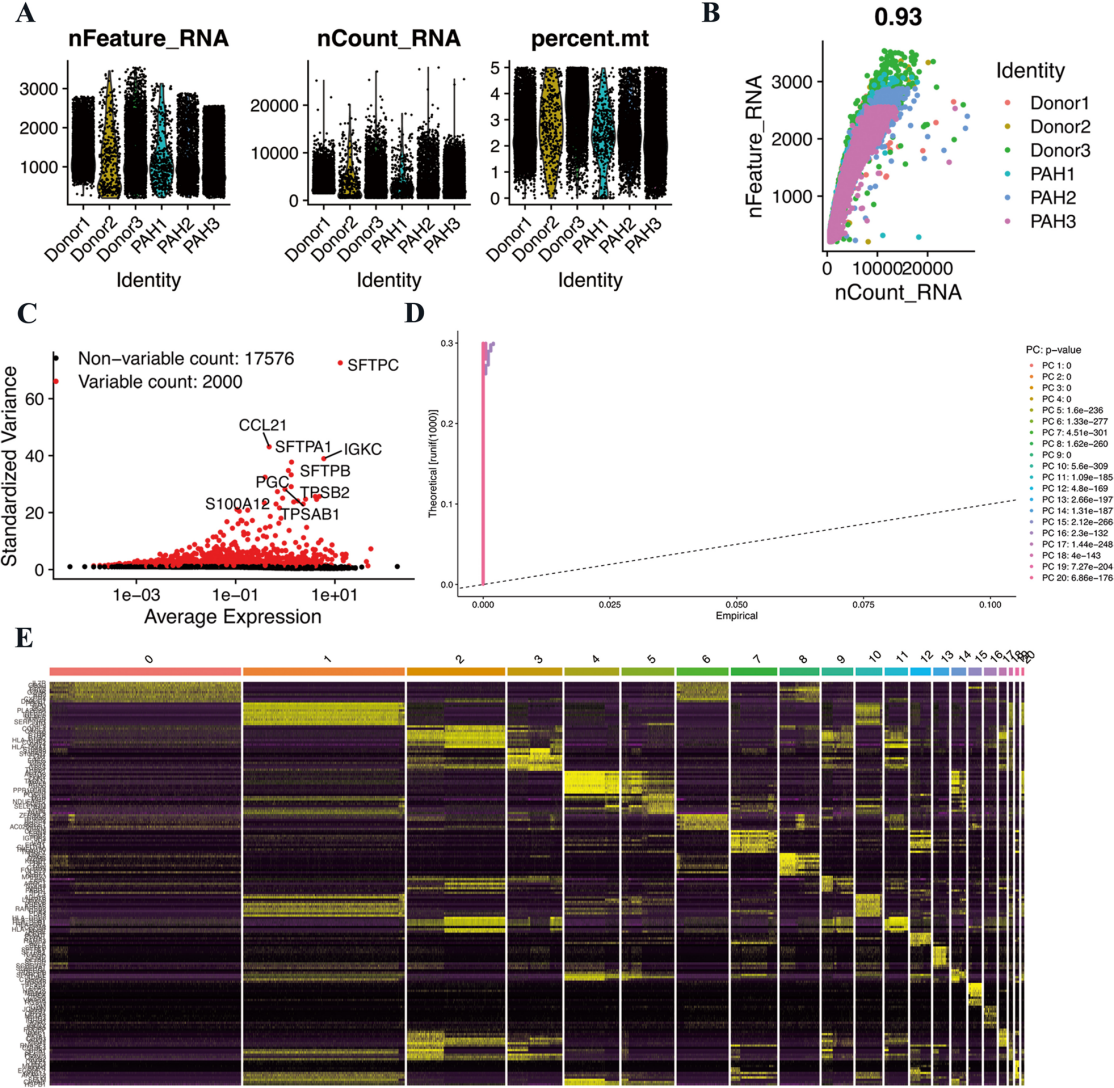
Single-cell RNA sequencing analysis of GSE210148 in PAH. **A** The features, counts, and mitochondrial gene percentage of each sample. **B** Correlation between genes and counts in each sample. **C** HVGs were colored in red, and the top 10 HVGs were labeled. **D** PCs selection using JackStraw function. **E** Heatmap of top 10 DEGs in each cluster. The top 10 DEGs were labeled in yellow color

The scRNA-seq data of GSE210248 was downloaded from the GEO database and analyzed through R software. In general, 42,280 cells comprising 19,576 cells from donors (control) and 22,704 cells from PAH patients were included. After filtrating improper gene amounts or mitochondrial genes ≥ 5%, 19,576 cells from donors and 21,896 cells from PAH patients remained. Figure 1A presented the expression characteristics of each sample. As shown in Fig. 1B, nCount_RNA (the number of unique molecular identifiers) was positively correlated with nFeature_RNA (the number of genes) with a correlation coefficient of 0.93. Figure 1C displayed and labeled the top 10 HVGs: SFTPC, CCL21, SFTPA1, IGKC, STFPA2, STFPB, PGC, TPSB2, TPSAB1, S100A12. The top 20 PCs identified by PCA were visualized by JackStrawPlot (Fig. 1D). In addition, the top 10 DEGs in each cluster were presented by heatmap and labeled in yellow (Fig. 1E).

### Cell clusters identification in scRNA-seq

**Fig. 2:**
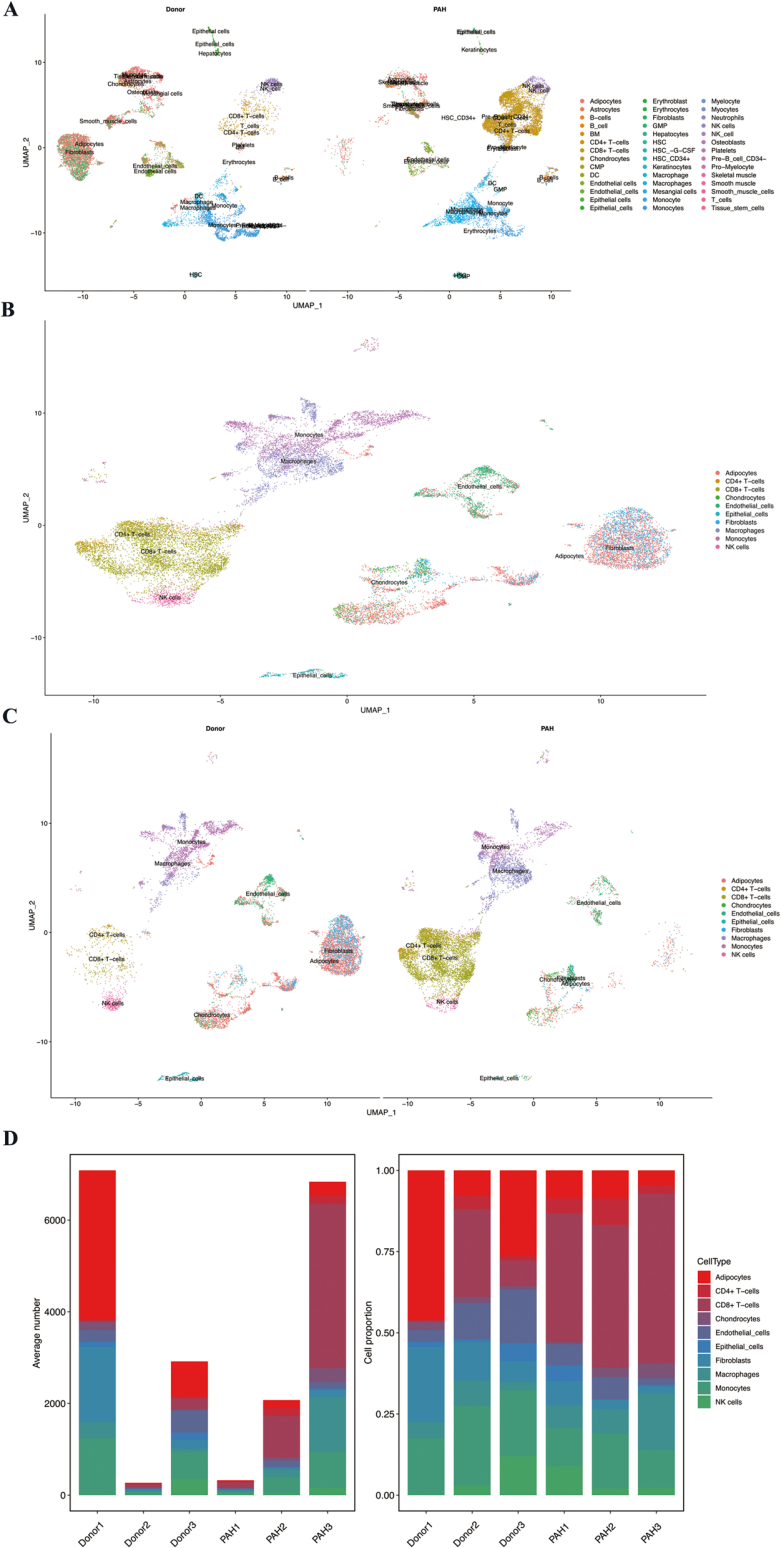
Clustering and annotation of single-cell RNA sequencing data. **A** UMAP visualization of PAH and donor groups. **B** UMAP visualization for the top 10 cell clusters. **C** UMAP visualization for the top 10 cell clusters in PAH and donor group. **D** Cluster distribution with the average cell number and cell proportion in each sample

Forty-one thousand four hundred seventy-two cells were divided into 42 cell clusters and visualized through UMAP, and cell annotation was performed by SingleR package (Fig. 2A). The number of cells in some clusters was too small, therefore, 10 clusters (Adipocytes, CD4+ T cells, CD8+ T cells, chondrocytes, endothelial cells, epithelial cells, fibroblasts, macrophages, monocytes, and NK cells) with the top 10 cell amounts were selected for subsequent analysis. Cell numbers of each cluster were shown in Table 2. The distribution of each cluster in the selected 10 clusters was presented in Fig. 2B, and the results of cell cluster distribution grouped by control and PAH were displayed in Fig. 2C. Additionally, the number and proportion of cells in each sample were exhibited in Fig. 2D. In comparison with the control group, the proportion of adipocytes (39.4% vs. 5.6%) and fibroblasts (18.1% vs. 2.5%) was significantly reduced in the PAH group, while CD8+ T cell (3.3% vs. 50.0%) and macrophages (4.3% vs. 14.8%) were notably increased in PAH lung tissues compared with donors.

**Table 2:** Cell numbers in each cluster

Sample	Donor 1	Donor 2	Donor 3	PAH 1	PAH 2	PAH 3
Adipocytes	3260	21	769	28	179	309
CD4+ T-cells	22	11	40	15	169	179
CD8+ T-cells	32	73	231	129	912	3579
Chondrocytes	171	5	29	2	60	310
Endothelial cells	255	30	484	21	145	139
Epithelial cells	114	2	162	16	0	31
Fibroblasts	1642	32	187	24	60	150
Macropages	348	21	74	23	156	1190
Monocytes	1221	66	592	38	351	789
NK cells	20	8	350	29	43	161

### DEGs of each cluster in GSE210248

**Fig. 3:**
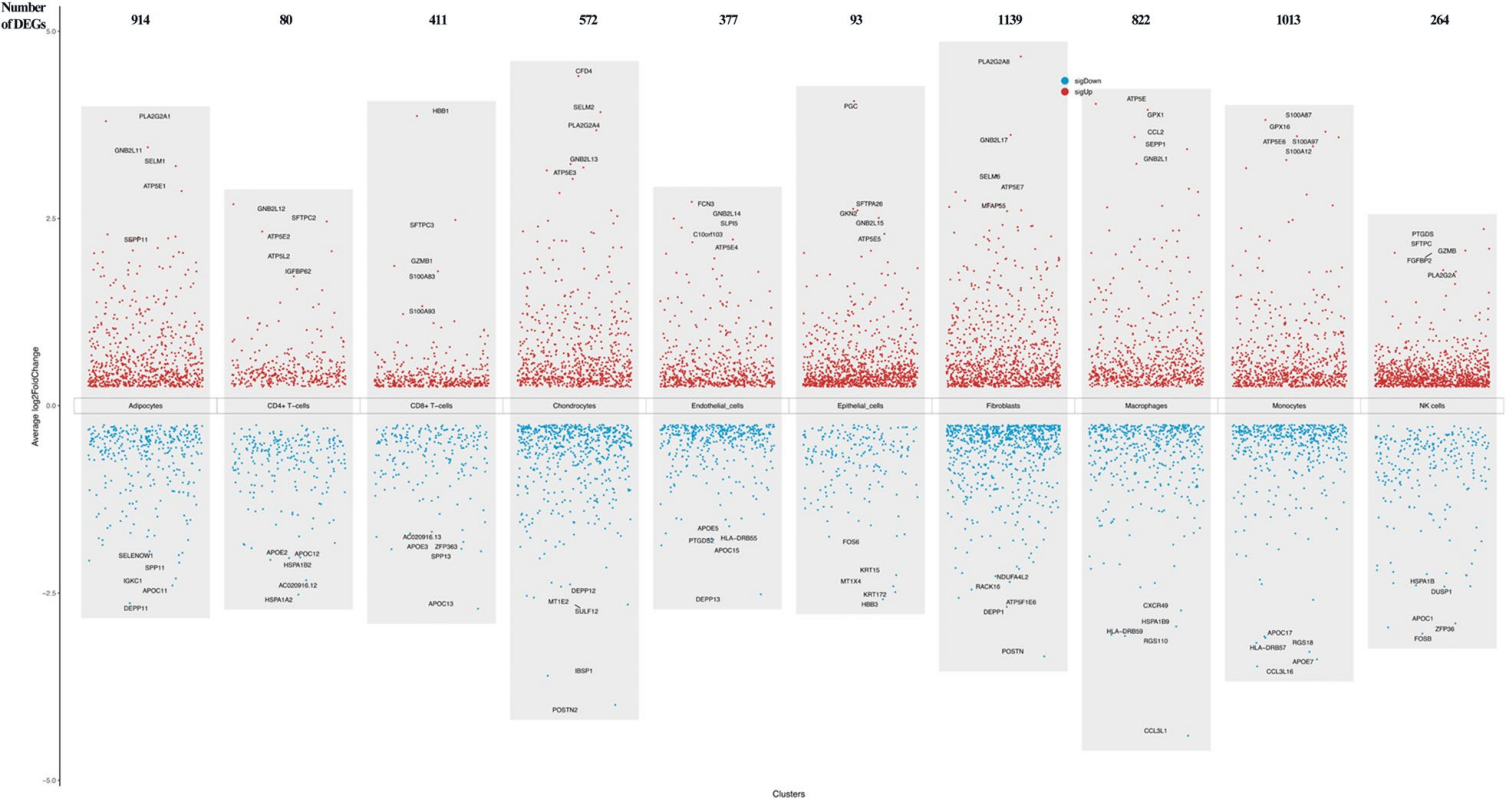
The top 5 upregulated and top 5 downregulated DEGs in the PAH group compared with the control group using the jjVolcano map

The DEGs of each cluster between the control and PAH groups were identified using the FindMarkers function. The top 10 DEGs in each cluster were listed in Table 3. For instance, GZMB, ATP5E, GNB2L17, GKN2, ATP5E6, SLPI5, SELM2, SFTPC3, SFTPC2, and GNB2L11 were the most significant DEG in NK cells, macrophages, fibroblasts, monocytes, epithelial_cells, endothelial_cells, chondrocytes, CD8+ T-cells, CD4+ T-cells, and adipocytes based on adjusted *p*-value. scRNAtoolVis package was further performed to intuitively illustrate the top 5 upregulated and the top 5 downregulated genes in the PAH group compared with the control group and visualized by jjvolcano (Fig. 3).

**Table 3:** The top 10 DEGs in each cell cluster between control and PAH group

cluster	p_val	avg_log2FC	pct.1	pct.2	p_val_adj	gene
NK cells	3.23E-57	2.07039378	0.952	0.339	6.32E-53	GZMB
NK cells	4.92E-50	2.0951293	0.722	0.064	9.63E-46	SFTPC
NK cells	1.60E-46	2.04057065	0.78	0.133	3.14E-42	FGFBP2
NK cells	8.08E-34	1.62372396	0.738	0.202	1.58E-29	SPON2
NK cells	5.62E-29	1.46648213	0.759	0.292	1.10E-24	HOPX
NK cells	2.47E-22	1.79013003	0.347	0.009	4.83E-18	S100A8
NK cells	4.38E-21	1.50916294	0.392	0.039	8.57E-17	S100A9
NK cells	1.65E-20	2.35866194	0.37	0.039	3.23E-16	PTGDS
NK cells	4.32E-19	1.80928128	0.286	0	8.45E-15	PLA2G2A
NK cells	4.38E-08	1.38693313	0.799	0.742	0.00085751	CCL3
Macrophages	4.67E-280	4.03072278	0.77	0	9.15E-276	ATP5E
Macrophages	4.20E-278	3.95218871	0.765	0	8.22E-274	GPX1
Macrophages	2.48E-270	3.22972958	0.747	0	4.85E-266	GNB2L1
Macrophages	2.95E-250	2.89671615	0.7	0	5.78E-246	ATP5L
Macrophages	5.45E-239	2.66855548	0.673	0	1.07E-234	C14orf2
Macrophages	2.78E-223	2.64832443	0.634	0	5.45E-219	TCEB2
Macrophages	2.08E-137	3.4251223	0.411	0	4.07E-133	SEPP1
Macrophages	1.86E-113	3.58695812	0.53	0.07	3.65E-109	CCL2
Macrophages	9.23E-66	2.85414508	0.359	0.053	1.81E-61	FABP4
Macrophages	3.97E-43	2.54189771	0.474	0.202	7.78E-39	CD524
Fibroblasts	2.28E-100	3.61565966	0.845	0	4.46E-96	GNB2L17
Fibroblasts	3.82E-92	4.6633342	0.863	0.077	7.47E-88	PLA2G2A8
Fibroblasts	8.51E-92	3.07543157	0.804	0	1.67E-87	SELM6
Fibroblasts	1.74E-90	2.69884577	0.948	0.244	3.42E-86	CFD8
Fibroblasts	1.34E-87	2.85203685	0.783	0	2.62E-83	ATP5E7
Fibroblasts	4.21E-73	2.7374571	0.827	0.179	8.24E-69	MFAP55
Fibroblasts	7.35E-72	2.68411819	0.812	0.145	1.44E-67	SLPI8
Fibroblasts	1.74E-68	2.59723614	0.831	0.192	3.41E-64	APOD6
Fibroblasts	4.53E-61	2.65393169	0.745	0.141	8.87E-57	RARRES1
Fibroblasts	3.16E-44	2.60853672	0.535	0.026	6.18E-40	HAS1
Monocytes	7.36E-248	3.58327494	0.634	0	1.44E-243	ATP5E6
Monocytes	4.65E-239	3.28083274	0.618	0	9.10E-235	GNB2L16
Monocytes	1.03E-232	3.65768818	0.607	0	2.02E-228	GPX16
Monocytes	7.81E-213	2.67403707	0.569	0	1.53E-208	ATP5L6
Monocytes	1.44E-185	2.47998988	0.514	0	2.82E-181	C14orf26
Monocytes	8.57E-169	3.59846333	0.763	0.311	1.68E-164	S100A97
Monocytes	5.30E-140	3.81550818	0.639	0.191	1.04E-135	S100A87
Monocytes	2.25E-111	3.17127943	0.343	0	4.40E-107	CCL3L31
Monocytes	1.87E-75	2.81840302	0.313	0.042	3.65E-71	CCL26
Monocytes	6.94E-71	3.46351087	0.332	0.063	1.36E-66	S100A12
Epithelial_cells	8.08E-16	2.60617797	0.712	0	1.58E-11	GKN2
Epithelial_cells	1.49E-15	4.06737565	0.77	0.085	2.92E-11	PGC
Epithelial_cells	3.32E-13	2.03992994	0.845	0.298	6.49E-09	NAPSA
Epithelial_cells	1.34E-12	2.62868014	0.809	0.404	2.62E-08	SFTPA26
Epithelial_cells	1.90E-12	1.91495198	0.835	0.255	3.73E-08	SFTPD
Epithelial_cells	2.83E-11	2.06833734	0.835	0.383	5.55E-07	SFTPA15
Epithelial_cells	1.58E-07	2.50721409	0.406	0	0.0030941	GNB2L15
Epithelial_cells	3.73E-07	2.29401564	0.388	0	0.0073072	ATP5E5
Epithelial_cells	7.30E-07	1.83558137	0.374	0	0.01428215	ATP5L5
Epithelial_cells	2.25E-05	1.98895129	0.295	0	0.4404569	FGG
Endothelial_cells	8.84E-45	2.37517579	0.525	0.056	1.73E-40	SLPI5
Endothelial_cells	2.61E-28	2.49810917	0.316	0	5.11E-24	GNB2L14
Endothelial_cells	6.41E-28	2.02900096	0.352	0.023	1.26E-23	S100A85
Endothelial_cells	6.67E-28	2.18205705	0.312	0	1.31E-23	ATP5E4
Endothelial_cells	1.61E-25	1.77403574	0.345	0.033	3.16E-21	S100A95
Endothelial_cells	1.60E-16	2.21981929	0.194	0	3.13E-12	C10orf103
Endothelial_cells	2.25E-13	1.96717524	0.157	0	4.40E-09	CA4
Endothelial_cells	5.31E-10	2.72216931	0.218	0.062	1.04E-05	FCN3
Endothelial_cells	2.81E-08	1.82344434	0.137	0.023	0.00054916	IL1RL1
Endothelial_cells	3.70E-08	1.78576199	0.147	0.03	0.00072335	HPGD
Chondrocytes	4.41E-91	3.92030676	0.815	0	8.64E-87	SELM2
Chondrocytes	1.11E-89	3.22559095	0.805	0	2.17E-85	GNB2L13
Chondrocytes	7.48E-85	3.18022527	0.771	0	1.46E-80	ATP5E3
Chondrocytes	3.46E-76	3.02854454	0.707	0	6.77E-72	PRKCDBP2
Chondrocytes	2.07E-70	2.8402306	0.663	0	4.04E-66	SEPW12
Chondrocytes	1.17E-61	4.40187507	0.722	0.075	2.29E-57	CFD4
Chondrocytes	1.89E-52	3.67716374	0.527	0.003	3.69E-48	PLA2G2A4
Chondrocytes	2.62E-33	3.14176007	0.512	0.083	5.13E-29	FBLN13
Chondrocytes	1.80E-16	2.53079619	0.288	0.051	3.52E-12	SFRP22
Chondrocytes	8.36E-15	2.6062608	0.195	0.013	1.64E-10	S100A84
CD8+ T-cells	6.49E-256	2.48007975	0.628	0.061	1.27E-251	SFTPC3
CD8+ T-cells	6.42E-217	1.79380393	0.318	0.01	1.26E-212	S100A83
CD8+ T-cells	2.04E-147	1.2227903	0.202	0.005	4.00E-143	PLA2G2A3
CD8+ T-cells	6.46E-73	1.12580015	0.265	0.037	1.26E-68	SCGB1A13
CD8+ T-cells	2.84E-66	1.32786131	0.298	0.052	5.56E-62	S100A93
CD8+ T-cells	9.02E-46	1.04286872	0.369	0.105	1.77E-41	PRF11
CD8+ T-cells	7.02E-43	1.86545565	0.336	0.1	1.38E-38	GZMB1
CD8+ T-cells	8.10E-30	1.01438835	0.301	0.1	1.58E-25	CFD3
CD8+ T-cells	2.45E-19	1.10154655	0.595	0.39	4.80E-15	NKG71
CD8+ T-cells	0.00369386	3.86973914	0.113	0.068	1	HBB1
CD4+ T-cells	9.14E-39	2.45943115	0.63	0.052	1.79E-34	SFTPC2
CD4+ T-cells	8.64E-27	2.68921472	0.301	0	1.69E-22	GNB2L12
CD4+ T-cells	1.37E-25	2.32625235	0.288	0	2.68E-21	ATP5E2
CD4+ T-cells	3.31E-23	2.0635857	0.26	0	6.48E-19	ATP5L2
CD4+ T-cells	2.50E-17	1.55626068	0.192	0	4.89E-13	GLTSCR22
CD4+ T-cells	6.26E-17	1.37413653	0.233	0.008	1.23E-12	S100A82
CD4+ T-cells	2.66E-15	1.72645498	0.384	0.069	5.21E-11	IGFBP62
CD4+ T-cells	5.22E-15	1.31327107	0.164	0	1.02E-10	ATP5G22
CD4+ T-cells	2.95E-11	1.54092574	0.384	0.099	5.78E-07	DCN2
CD4+ T-cells	2.06E-10	1.35530821	0.356	0.091	4.02E-06	CFD2
Adipocytes	1.57E-180	3.44999227	0.76	0	3.08E-176	GNB2L11
Adipocytes	3.29E-166	3.7989569	0.83	0.128	6.44E-162	PLA2G2A1
Adipocytes	7.83E-165	3.19946236	0.721	0	1.53E-160	SELM1
Adipocytes	3.95E-160	2.86553955	0.709	0	7.73E-156	ATP5E1
Adipocytes	2.64E-127	2.25769098	0.617	0	5.17E-123	ATP5L1
Adipocytes	2.84E-125	2.2359181	0.611	0	5.56E-121	C14orf21
Adipocytes	6.09E-123	2.20576993	0.604	0	1.19E-118	TCEB21
Adipocytes	1.03E-99	2.28601276	0.527	0	2.01E-95	SEPP11
Adipocytes	6.50E-93	2.10602684	0.503	0	1.27E-88	PRKCDBP1
Adipocytes	1.39E-47	2.24942588	0.306	0	2.72E-43	C10orf101

### DEGs of pulmonary tissue in GSE113439

**Fig. 4:**
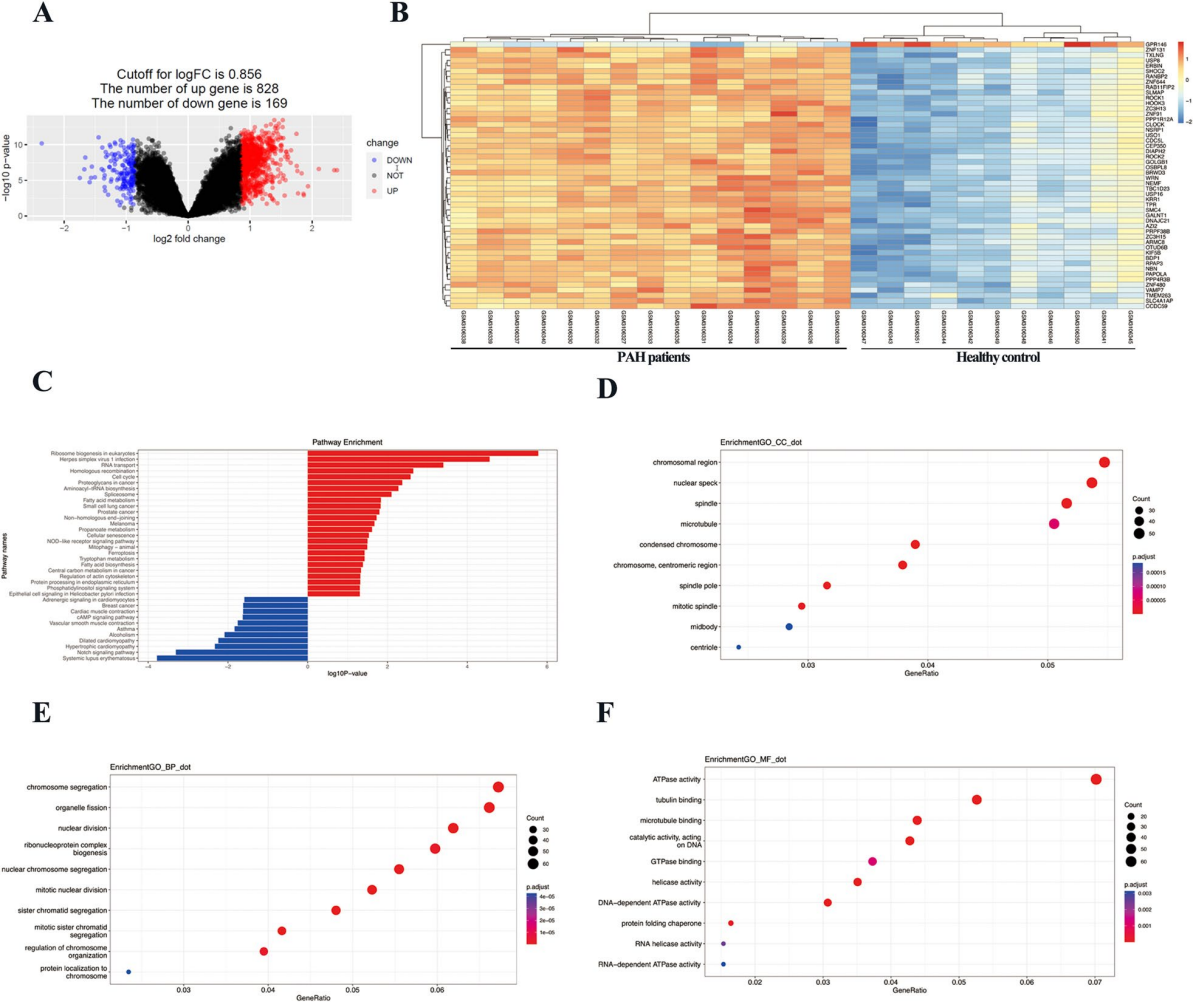
DEGs of lung tissue from GSE113439 dataset. **A** Volcano plot of DEGs with |log_2_FC|> 0.856 and adjusted *p* value < 0.05. Upregulated and downregulated genes were colored by red and blue, respectively. **B** Heatmap displaying the top 50 DEGs of GSE113439. **C** KEGG of DEGs in GSE113439. Dot blot of the top 10 CC (**D**), BP (**E**), and MF (**F**) pathways of GO in GSE113439. The size and color of dots represent the count of genes and adjusted *p* value in the selected pathway

The limma package was utilized to explore the DEGs in lung samples of 11 control and 15 PAH patients. DEGs with |logFC|> 0.856 and adjusted *p*-value < 0.05 were presented in Fig. 4A. Compared with the control group, 828 genes were found upregulated, and 169 genes were downregulated in the lung tissue of PAH patients. A Heatmap of the top 50 DEGs was shown in Fig. 4B. The majority of DEGs were upregulated, only the gene GPR146 was found downregulated among these top 50 DEGs. The results of KEGG functional enrichment analysis were shown in Fig. 4C. These upregulated DGEs were enriched in ribosome biogenesis in eukaryotes, herpes simplex virus 1 infection, RNA transport, homologous recombination, cell cycle, proteoglycans in cancer, aminoacyl-tRNA biosynthesis, spliceosome, fatty acid metabolism, small cell lung cancer, etc. The downregulated DEGs were enriched in systemic lupus erythematosus, Notch signaling pathway, hypertrophic cardiomyopathy, alcoholism, asthma, vascular smooth muscle constriction, cAMP signaling pathway, cardiac muscle contraction, breast cancer, and adrenergic signaling in cardiomyocytes. The cell component (CC), biological process (BP), and molecular function (MF) of GO enrichment analysis were presented in Fig. 4D-F. The top 10 enriched pathways in CC included chromosomal region, nuclear speck, spindle, microtubule, condensed chromosome, chromosome, centromeric region, spindle pole, mitotic spindle, midbody, and centriole; The top 10 enriched pathways in BP included chromosome segregation, organelle fission, nuclear division, ribonucleoprotein complex biogenesis, nuclear chromosome segregation, mitotic nuclear division, sister chromatid segregation, mitotic sister chromatid segregation, regulation of chromosome organization, protein localization to chromosome; The top 10 enriched pathways in MF included ATPase activity, tubulin binding, microtubule binding, catalytic activity, acting on DNA, GTPase binding, helicase activity, DNA-dependent ATPase activity, protein folding chaperone, RNA helicase activity, and RNA-dependent ATPase activity.

### Different immune cell infiltration of pulmonary tissue in GSE113439

**Fig. 5:**
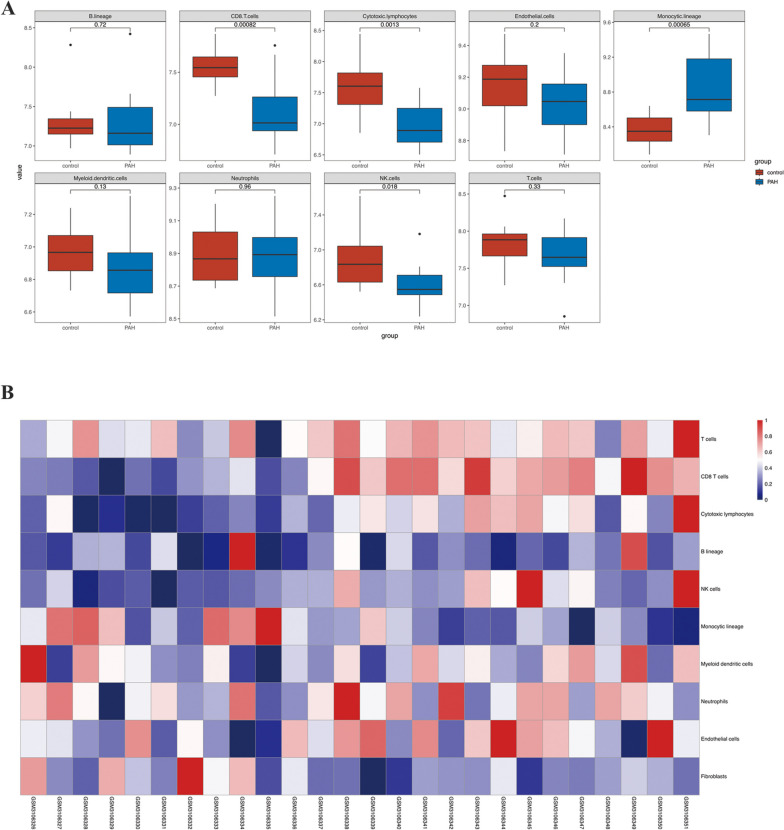
Dysregulated immune cells infiltration in PAH lungs. **A** The box plot of immune cells abundance in control and PAH group. **B** Heatmap displaying the abundance of immune cells in each sample of lung tissues in control and PAH patients

Using microarray transcriptome data from GSE113439, the MCP counter was utilized to evaluate the immune cell infiltration in control and PAH lung samples. As shown in Fig. 5A, statistically decreased distribution of CD8+ T cells, cytotoxic lymphocytes, and NK cells were found in lung tissues of PAH patients compared with control subjects. However, increased infiltration of monocytic lineage was found in PAH lung tissue. The Heatmap further displayed the abundance of each cell type with normalization value ranging from 0–1 in each sample between the control and PAH group (Fig. 5B) (PAH: GSM310626-GSM3106340; Control: GSM3106341-GSM3106351).

### Protein-protein interaction network (PPI) network and common DEGs identification in GSE210248 and GSE113439

**Fig. 6:**
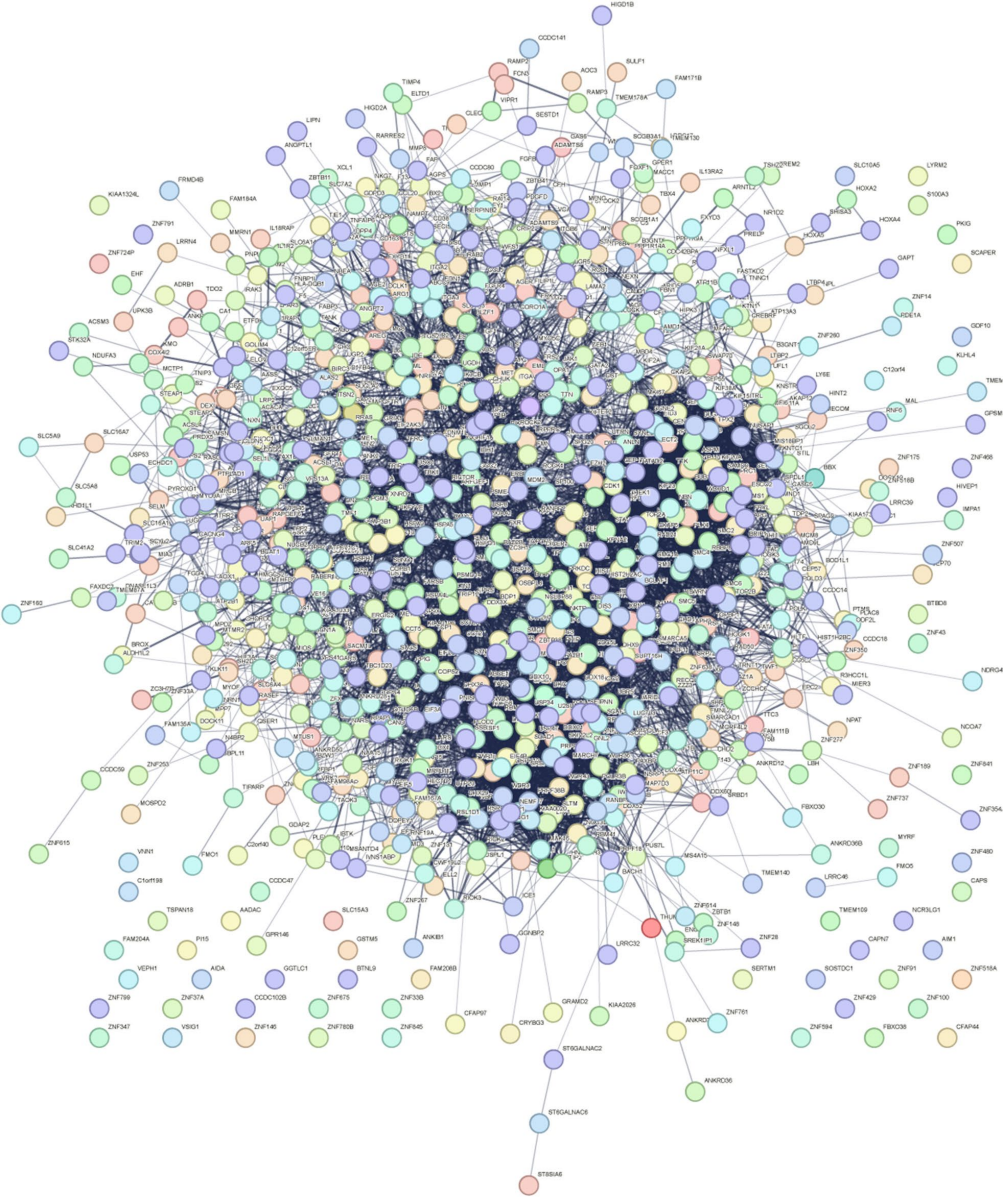
PPI network of DEGs from GSE113439

**Fig. 7:**
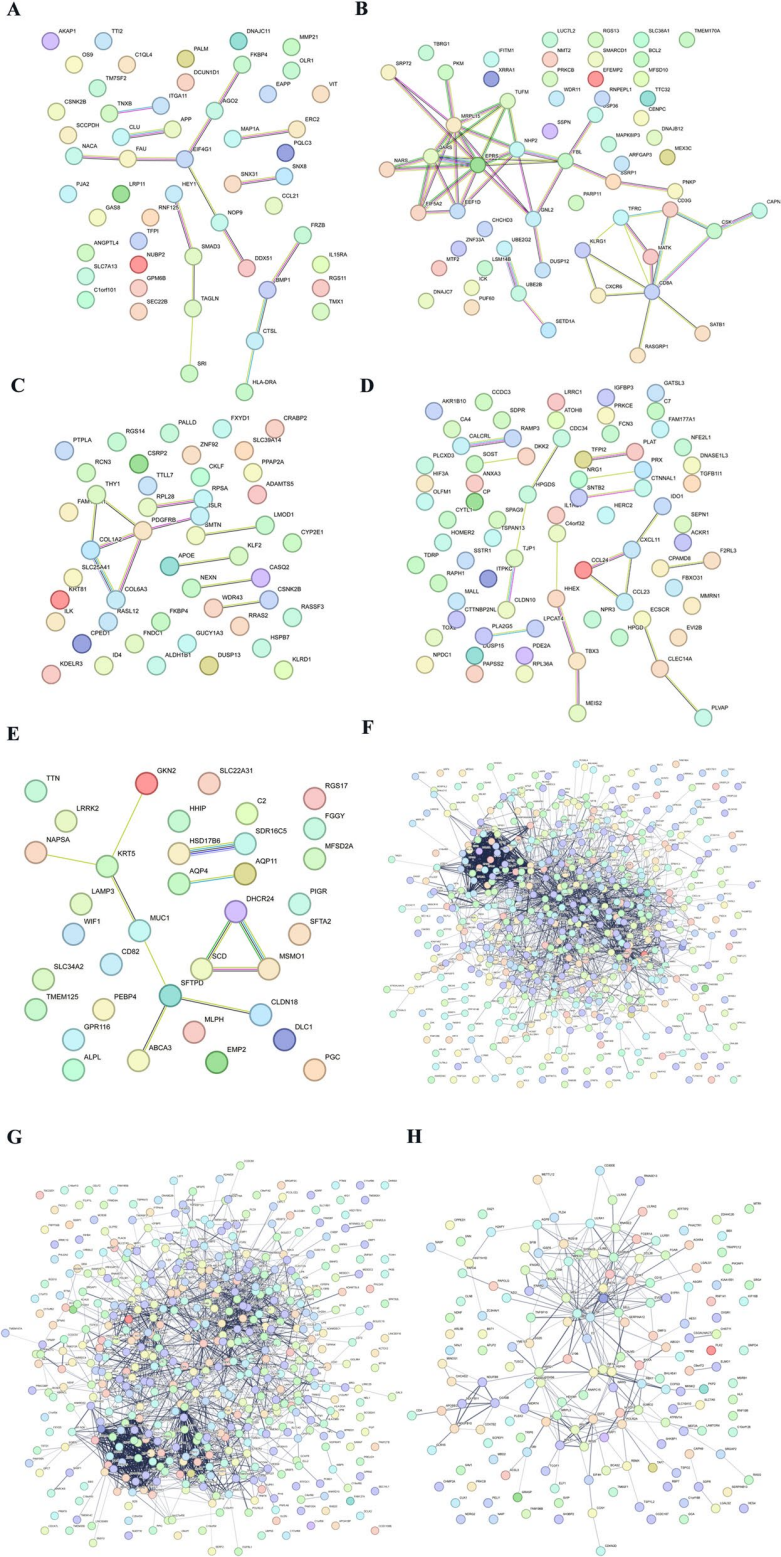
PPI network of DEGs in each cluster of GSE210248. PPI network of the DEGs in adipocytes (**A**), CD8+ T cells (**B**), chondrocytes (**C**), endothelial cells (**D**), epithelial cells (**E**), fibroblasts (**F**), macrophages (**G**), and monocytes (**H**)

**Fig. 8:**
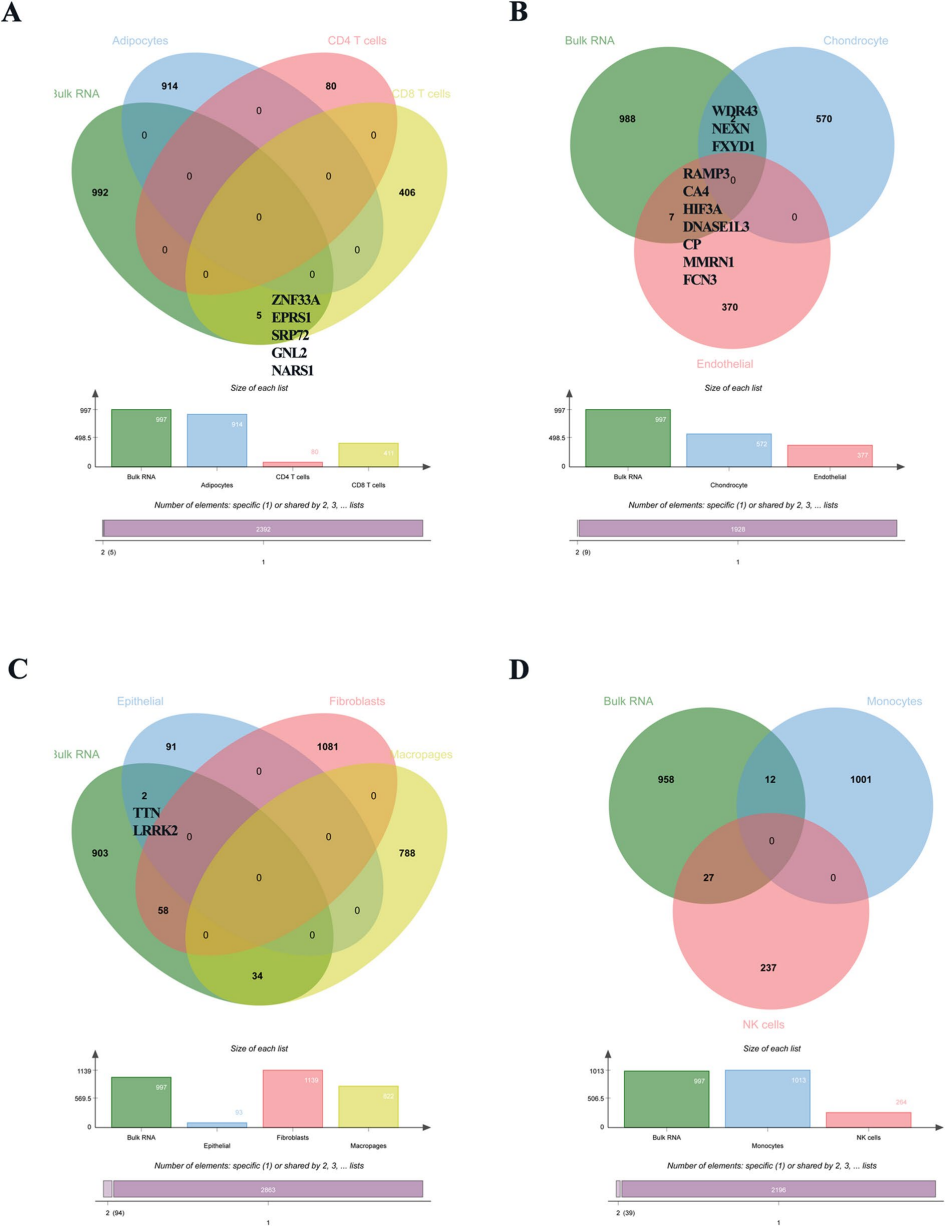
Veen’s diagram showing the common DEGs from GSE210248 and DEGs from GSE113439. **A** Veen’s diagram showing the common DEGs from GSE113439, DEGs from adipocytes, CD4+ T cells, and CD8+ T cells in GSE210248. **B** Veen’s diagram showing the common DEGs from GSE113439, DEGs from chondrocytes, and endothelial cells in GSE210248. **C** Veen’s diagram showing the common DEGs from GSE113439, DEGs from epithelial cells, fibroblasts, and macrophages. **D** Veen’s diagram showing the common DEGs from GSE113439, DEGs from monocytes and NK cells

The PPI network of DEGs from GSE113439 was generated by STRING (Fig. 6). The PPI network consisted of 945 nodes and 7266 edges in 997 DEGs. Then, the PPI network of the DEGs in each cluster of GSE210248 was constructed through the STRING online website. Figures 7A and 8H presented the PPI network of 914 DEGs in adipocytes, 411 DEGs in CD8+ T cells, 572 DEGs in chondrocytes, 377 DEGs in endothelial cells, 93 DEGs in epithelial cells, 1139 DEGs in fibroblasts, 822 DEGs in macrophages, and 1013 DEGs in monocytes with the adjusted *p*-value < 0.05. A Venn diagram was drawn to screen out the common hub genes from GSE210248 and GSE113439. As shown in Fig. 8, a series of genes were identified through overlapping DEGs from each cluster in GSE210248 and DEGs from GSE113439. The details of overlapped genes were listed in Table 4.

**Table 4:** Common DEGs from each cluster of GSE210248 and GSE113439

List 1	List 2	Common DEGs
DEGs from GSE113439	DEGs from CD8+ T cells in GSE210248	ZNF33A, EPRS1, SRP72, GNL2, NARS1
DEGs from GSE113439	DEGs from Chondrocytes in GSE210248	WDR43, NEXN, FXYD1
DEGs from GSE113439	DEGs from Endothelial cells in GSE210248	RAMP3, CA4, HIF3A, DNASE1L3, CP, MMRN1, FCN3
DEGs from GSE113439	DEGs from Epithelial cells in GSE210248	TTN, LRRK2,
DEGs from GSE113439	DEGs from Fibroblasts in GSE210248	SMC4, CRIP2, STK38L, EPS8, MTREX, ST6GALNAC6, SEMA3B, C1orf198, FRMD4B, HIGD2A, DEPP1, RAMP2, CFH, NUCB2, CFI, MORF4L2, ITGA3, PPP1R14A, LAMA2, LTBP2, FAT1, CALD1, LBH, ANKRD36C, POSTN, PLS3, TMEM204, MTHFD2, ANO1, ARID5B, LRRC32, PDE1A, BST2, AOC3, IGF1, TSHZ2, PDGFD, MAP1B, ANK2, TXNRD1, SHISA3, ANGPTL1, FBN1, UGDH, PRELP, FGF7, AKAP12, SLC16A7, NAMPT, FAP, SULF1, STEAP2, LRRC17, STEAP1, HP, AOX1, HAS2, TNFAIP6
DEGs from GSE113439	DEGs from Macrophages in GSE210248	PRPF38B, RBPJ, SNX2, MIS18BP1, FCHO2, RARRES2, GOLIM4,. CLTC, WASHC4, SWAP70, TOP1, EIF5, PTMS, SP100, SELENOM, IFI16, MRC1, FMN1, FILIP1L, TLR2, SLC1A3, TIPARP, ACSL1, VCAN, MFAP4, USP53, ANKRD22, ELL2, SCGB3A1, IL1RAP, F13A1, UAP1, B3GNT5, CCDC80
DEGs from GSE113439	DEGs from Monocytes in GSE210248	AZI2, YME1L1, HSPD1, BBX, HSPA5, ATP6V1A, HIF1A, RASGEF1B, FCER1A, PKP2, AQP9, SERPINB2
DEGs from GSE113439	DEGs from NK cells in GSE210248	USP16, KIF5B, SLTM, GOLGA4, ICAM2, EIF3A, S100A4, PRDX5, RIOK3, EIF2S2, NCL, SYNE1, SYNE2, JAK1, HSPH1, XCL1, CCL5, NKG7, PLAC8, GNLY, FGFBP2, SPP1, AREG, CXCL8, PLA2G2A, HLA-DQB1, SCGB1A1

### Hub genes identification in PAH

**Fig. 9:**
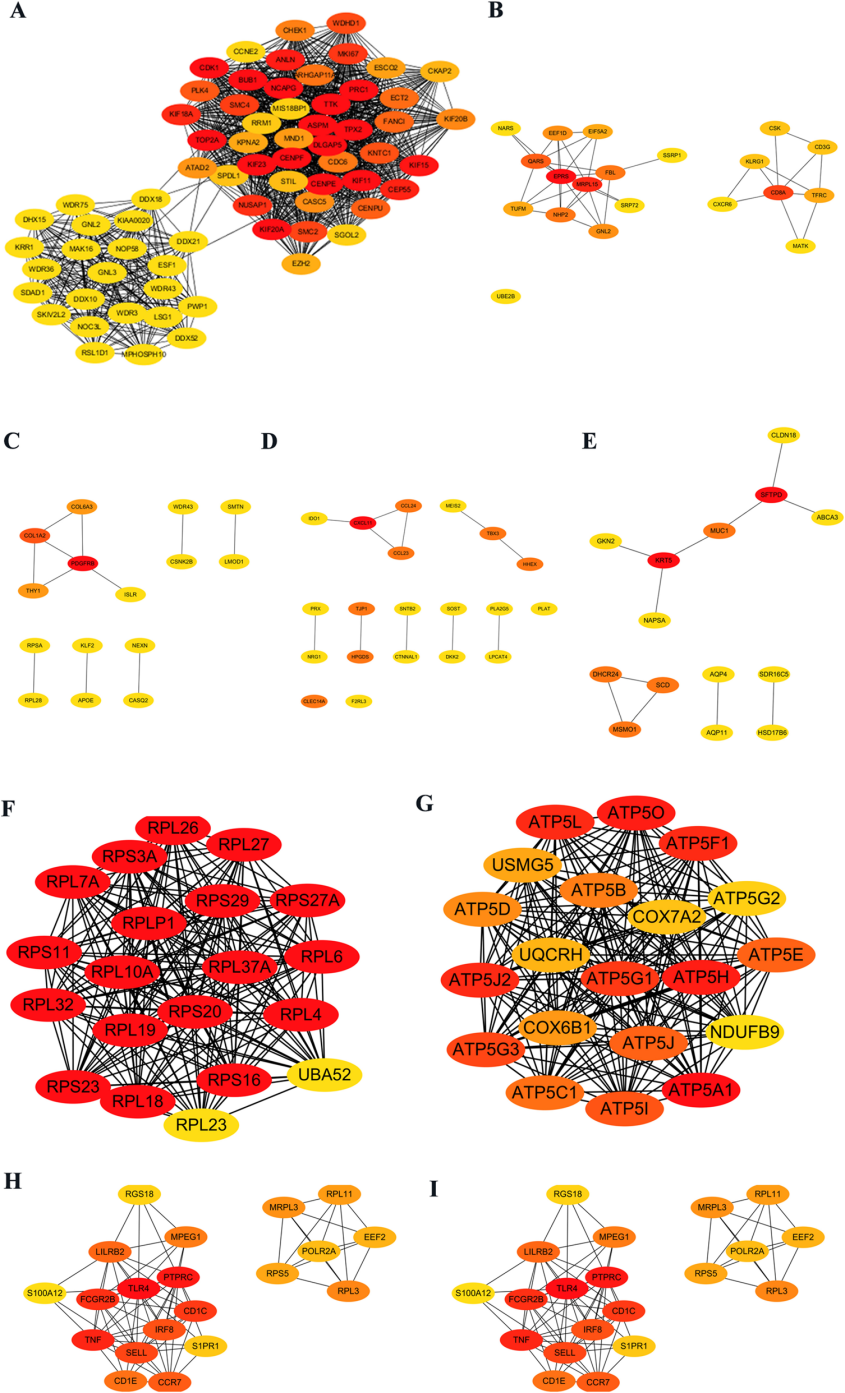
Hub genes of GSE113439 and hub genes of each cluster from GSE210248. **A** Selected hub genes in module 1 of GSE113439 using MCODE pulg-in. The Top 20 hub genes in CD8+ T cells (**B**), chondrocytes (**C**), endothelial cells (**D**), epithelial cells (**E**), fibroblasts (**F**), macrophages (**G**), monocytes (**H**), and NK cells (**I**) identified by cytohubba plug-in according to nodes’ score by MCC method from GSE210248

**Fig. 10:**
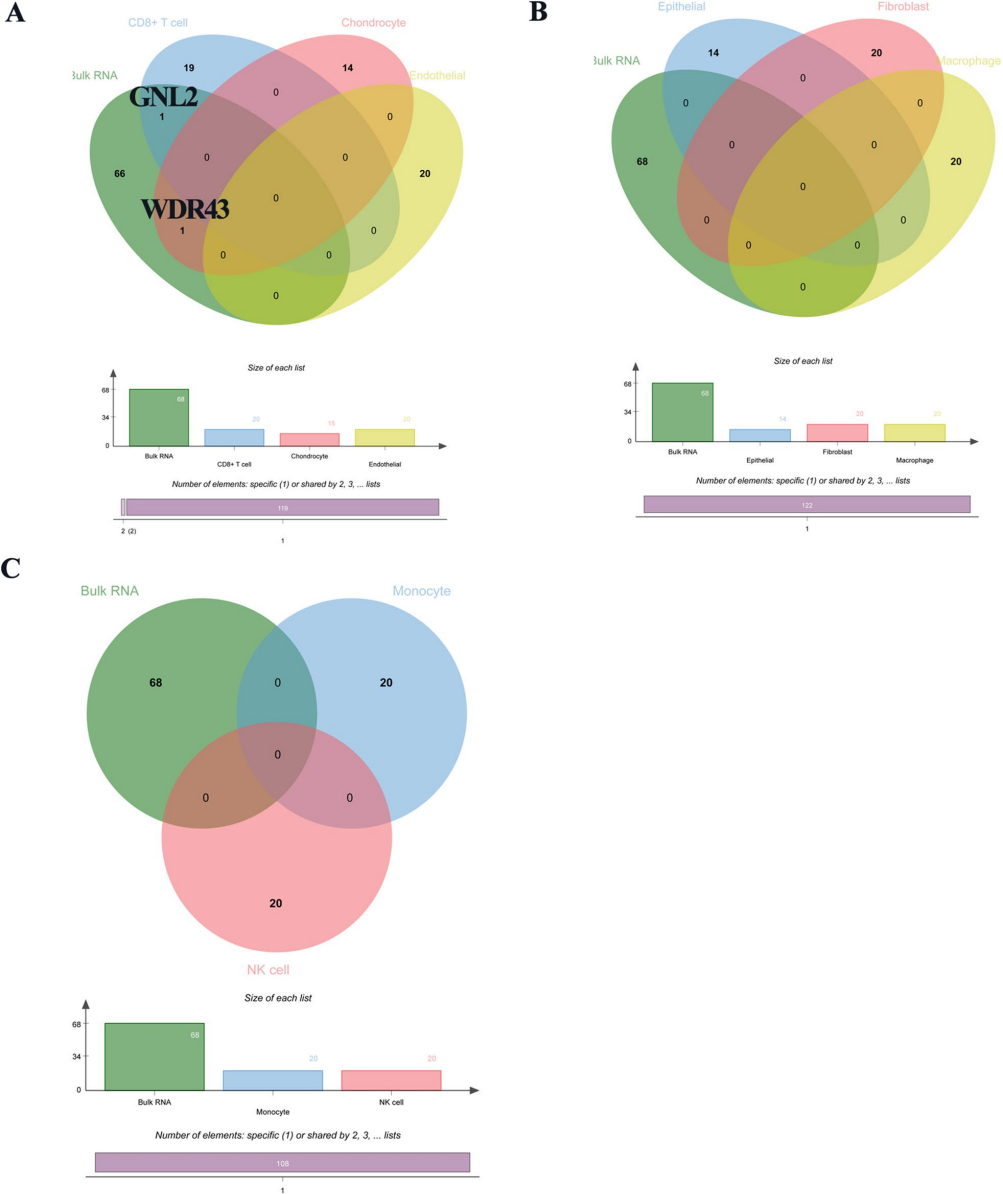
Veen’s diagram showing the common hub genes from GSE210248 and GSE113439. **A** Veen’s diagram showing the common hub genes from GSE113439, CD8+ T cells, chondrocytes, and endothelial cells from GSE210248. **B** Veen’s diagram showing the common hub genes from GSE113439, epithelial cells, fibroblasts, and macrophages from GSE210248. **C** Veen’s diagram showing the common hub genes from GSE113439, monocytes and NK cells from GSE210248

**Fig. 11:**
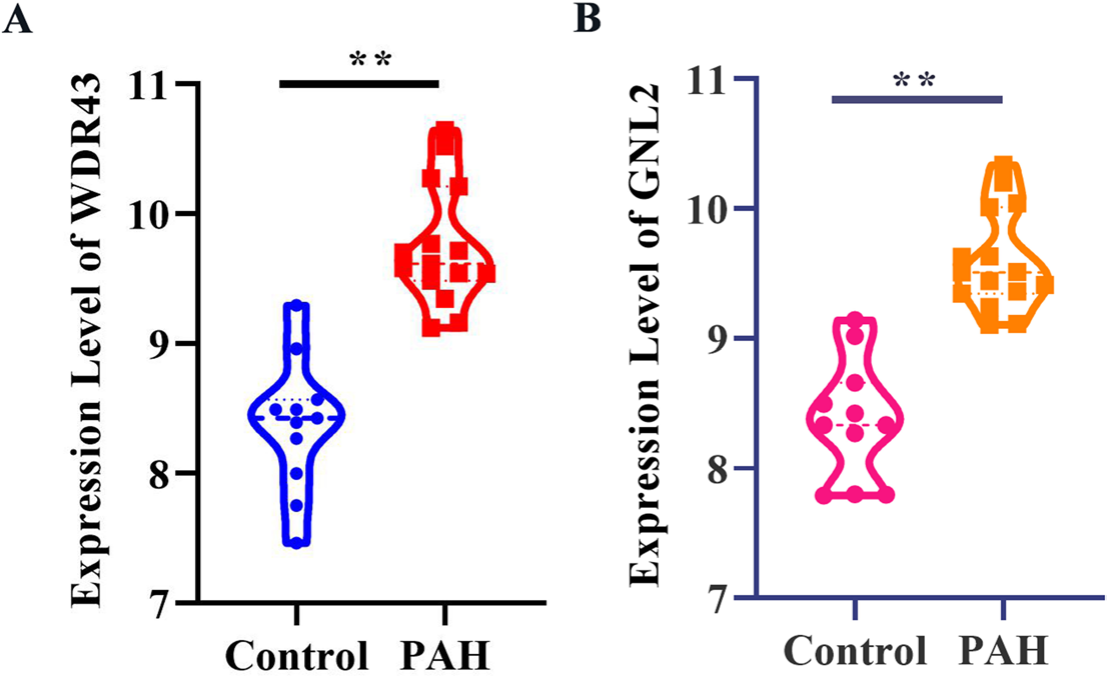
The expression of selected hub genes. The expression of WDR43 (**A**) and GNL2 (**B**) in lung samples of 11 healthy control and 15 PAH patients in GSE113439. **p < 0.01

Considering the amounts of selected common DEGs were relatively large. MCODE was utilized for the selection of candidate hub genes from the PPI network of 997 DEGs in GSE113439. Module 1 with the highest score (68 nodes and 1142 edges) was screened out (Fig. 9A). The centralities of the candidate genes in module 1 were evaluated by the CentiScaPe plug-in and the details were shown in Table 5. Additionally, the CytoHubba plug-in was used for ranking nodes in module 1 using MCC methods. The MCODE score of each gene is also summarized in Table 5. Cytohubba plug-in was performed to further simplify these hub genes and pick out the most critical genes using the MCC method. No overlapped genes were found between DEGs from adipocytes, CD4+ T cells, and DEGs from GSE113409. Therefore, the top 20 genes were filtered out in the remaining 8 clusters of GSE210248 and presented in Fig. 9B-I. Less than 20 DEGs in chondrocytes and epithelial cells were presented because there are only 15 DEGs in chondrocytes and 14 DEGs in epithelial cells were included in the network of Cytoscape software. We further screened out the common hub genes using data from module 1 and the top 20 genes in these clusters. As shown in Fig. 10, WDR43 in chondrocytes and GNL2 in CD8+ T cells were finally identified as the most significant genes in PAH. Furthermore, we detected the expression of WDR43 and GNL2 in the lung samples of 15 PAH patients and 11 control subjects in GSE113439 and found significantly increased WDR43 and GNL2 expression (Fig. 11).

**Table 5:** The centralities and MCODE score of candidate genes evaluated by CentiScape and CytoHubba plug-in

Gene name	Betweenness	Centroid	Degree	MCODE_Score
KIF11	8.971739	-21	44	29.98578
GNL3	0	-43	22	24.85755
KIF23	355.464	-1	45	29.98578
NCAPG	8.971739	-21	44	29.98578
WDR43	0	-43	22	24.85755
DLGAP5	8.971739	-21	44	29.98578
MND1	0.232716	-35	31	30
CDC6	4.186865	-26	39	30.36032
ANLN	7.778993	-22	43	29.98578
CDK1	8.971739	-21	44	29.98578
WDR36	0	-43	22	24.85755
ECT2	7.922387	-22	43	30.20871
NOC3L	0	-43	22	24.85755
KIF20A	8.971739	-21	44	29.98578
BUB1	8.971739	-21	44	29.98578
CEP55	7.976828	-22	43	29.98578
KRR1	0	-43	22	24.85755
WDR75	0	-43	22	24.85755
TOP2A	712.4563	1	46	29.98578
DHX15	0	-43	22	24.85755
CENPF	8.971739	-21	44	29.98578
WDR3	0	-43	22	24.85755
PRC1	8.971739	-21	44	29.98578
TPX2	8.971739	-21	44	29.98578
GNL2	0	-43	22	24.85755
NUSAP1	6.571843	-23	42	29.98578
TTK	8.971739	-21	44	29.98578
MAK16	0	-43	22	24.85755
RSL1D1	0	-43	22	24.85755
KIF15	7.384273	-22	43	29.98578
DDX21	544.1357	-22	24	24.85755
MKI67	6.977592	-23	42	29.98578
CENPU	4.789195	-25	40	30.77897
KIAA0020	0	-43	22	24.85755
KPNA2	511.8998	-11	35	28.93763
SMC2	6.635866	-23	42	29.98578
ARHGAP11A	3.674459	-26	39	30.40952
CHEK1	4.024379	-27	39	30.53109
KIF20B	336.0392	-7	39	29.4958
KNTC1	6.087509	-24	42	30.63529
SDAD1	0	-43	22	24.85755
FANCI	3.879142	-26	39	30.77897
ATAD2	2.40196	-30	36	29.93952
CKAP2	2.746163	-30	35	26.91765
STIL	0.375244	-37	30	28
WDHD1	1.914333	-28	37	30.20871
EZH2	0.376068	-37	30	29
DDX10	0	-43	22	24.85755
SMC4	5.440622	-24	41	29.98578
PLK4	4.681379	-26	40	30.63529
RRM1	1.342468	-34	31	26.81379
PWP1	0	-43	22	24.85755
SGOL2	0.191789	-37	29	27.8069
CASC5	2.253639	-30	36	29.82955
ASPM	8.971739	-21	44	29.98578
CCNE2	0.272622	-40	27	24.92877
MIS18BP1	0.4634	-36	30	26.59355
ESF1	292.2717	-23	23	24.85755
LSG1	0	-43	22	24.85755
MPHOSPH10	0	-43	22	24.85755
NOP58	219.4768	-23	23	24.85755
DDX18	669.6512	-22	24	24.85755
DDX52	0	-43	22	24.85755
ESCO2	3.293206	-28	37	29.87903
SPDL1	0.679996	-35	31	26.45565
CENPE	8.971739	-21	44	29.98578
KIF18A	6.389363	-23	42	29.98578
SKIV2L2	0	-43	22	24.85755

## Discussion

The present study for the first time indicated WDR43 and GNL2 might act as key genes involved in the pathogenesis of PAH, providing a novel potential underlying mechanism of PAH. In the current study, common DEGs were screened out using integrated analysis of scRNA-seq and microarray transcriptome through the limma package and Seurat package in R software. Subsequently, the PPI network of DEGs was constructed using the STRING website. Then, Cytoscape software was utilized to screen out the hub genes in the cluster of GSE210248 and GSE113439. Ultimately, we identified two hub genes (WDR43 and GNL2) in PAH through a series of bioinformatics analyses.

MCP counter illustrated dysregulated landscape of immune cells in lung tissues of PAH patients, which is consistent with previous reports. Marlene reviewed immune dysregulation in PAH and how immune-mediated vascular injury promoted PAH development [25]. For instance, circulating autoantibodies against endothelial cells might enhance the apoptosis of endothelial cells in PAH [26]. T cells and NK cells were considered as beneficial factors during the pathogenesis of PAH [27, 28]. Additionally, the role of perivascular macrophages has received extensive attention from researchers. Widespread Cd68+ macrophages were detected in occlusive plexiform lesions in clinical and experimental PAH models [29]. Inactivation or deletion of macrophages could prevent the development of PAH [30]. More researches need to be carried out to further explore the role of various immune cells in PAH and the underlying mechanisms.

The WD40 repeat (WDR) domain is the most abundant protein interaction domain in the human proteome. The WDR43 gene is located on chromosome 2 and encodes the WDR43 protein containing 677 amino [31]. Of note, WDR43 is an essential subunit of multiprotein complexes and is involved in a series of signaling pathways including ubiquitin-proteasome pathway, epigenetic regulation, DNA damage repair, and immune-related pathways [32]. For instance, the NOL11-WDR43-Cirhin protein complex is necessary for mitotic chromosome segregation [33]. Intriguingly, several bioinformatics analysis identified WDR43 as a crucial oncogene contributing to the development of colorectal/lung cancer via promoting the migration and proliferation of cancer cells through GEO and The Cancer Genome Atlas (TCGA) database. Mechanistically, c-MYC/WDR43/MDM2 mediated p53 degradation, and cyclin-dependent kinase 2 were involved in the underlyng mechanism [34–36]. However, the role of WDR43 in PAH remains uninvestigated.. Similarly, the imbalance of proliferation and apoptosis in pulmonary artery smooth muscle cells (PASMCs) was also the key characteristic in pulmonary hypertension [37]. Therefore, we speculate WDR43 might contribute to PASMCs proliferation and migration, then leading to the pulmonary artery remodeling.

GNL2, the G protein nucleolar 2, was found essential for cell growth and development through participating in the cell-cycle regulation pathway [38]. GNL2 acts as a checkpoint for ribosome export, and it plays a vital role in facilitating ribosomal biogenesis and protein synthesis [39]. GNL2 was found to play a critical role in the RNA metabolic network and was associated with proliferation [40]. Increased expression of GNL2 was correlated with poor prognosis in ovarian cancer patients with 1p34.3 amplifications [41]. Results from another scRNA-seq data of periodontitis revealed GNL2 was upregulated in T cells [42]. While the role of GNL2 in PAH and its potential underlying mechanisms needs further exploration. In combination with the KEGG analysis in the current study, GNL2 might participate in the underlying mechanism of PH through the influence on the biosome biogenesis and cell cycle.

Nowadays, high-throughput RNA sequencing has been widely used to explore novel mechanisms of PAH [6, 43]. Especially, with the rapid development of single-cell sequencing, integrated bioinformatics analysis of microarray transcriptome and scRNA-seq, a newly-rising research method, has attracted researchers’ attention lately [44]. A recent study indicated hpgd was a key gene in pulmonary artery endothelial cells (PAECs) using scRNA-seq data from PAECs of control and PAH rodents [45]. There still remains largely unknown on the mechanism of PAH through integrated bioinformatics analysis. The current research might provide a novel insight into the pathogenesis of PAH.

## Conclusion

In summary, we performed an integrated bioinformatics analysis of single-cell sequencing andmicroarray transcriptome. Multi-step analysis suggested that WDR43 and GNL2 were increased in PAH lung tissues and they were identified as hub genes in the pathogenesis of PAH. Our results highlight WDR43 and GNL2 as potential biomarkers and pharmacological therapeutic targets for PAH.

## Data Availability

Publicly available GEO datasets were analyzed in this study (https://www.ncbi.nlm.nih.gov/geo/) under the accession numbers GSE210248 and GSE113439.
